# The Mediating Effect of Self-Esteem on the Relationship Between Job Satisfaction, Leisure Satisfaction, and Quality of Life Among Korean Police Officers

**DOI:** 10.3390/healthcare12232389

**Published:** 2024-11-28

**Authors:** Seung-Woo Han, Hyun-Seok Yoon

**Affiliations:** 1 Department of Nursing, Kwangju Women’s University, Gwangsan-gu, Gwangju 62396, Republic of Korea; swhan@kwu.ac.kr; 2 Department of Police Administration, Kwangju Women’s University, Gwangsan-gu, Gwangju 62396, Republic of Korea

**Keywords:** leisure activities, relational dynamics mediation pathways, self-esteem, job satisfaction, quality of life, police

## Abstract

**Background/Objectives:** This study was to determine how self-esteem mediates the relationships between leisure satisfaction, job satisfaction, and quality of life (QoL) among Korean police officers. In addition, the purpose is to comprehensively understand the quality of life of police officers by confirming the influence of variables affecting the quality of life of police officers and the direct and indirect effects of each variable and to provide scientific basis data for the application of intervention programs to improve the quality of life. **Methods:** The study was conducted with police officers working in three police stations in K Metropolitan City and J Province from August 1 to 20, 2024. Data collected were analyzed using the PROCESS Macro (Model 6), which assessed the significance of indirect effects and the variations across mediation pathways. **Results:** When looking at factors affecting QoL, self-esteem had statistically significant direct and total effects (γ = 0.115, *p* = 0.001). Leisure satisfaction was found to have a statistically significant total effect (γ = 0.296, *p* < 0.001) due to the direct effect (γ = 0.273, *p* < 0.001) and indirect effect (γ = 0.023, *p* < 0.05) on QoL. In addition, job satisfaction was found to have a statistically significant total effect (γ = 0.450, *p* < 0.001) due to the direct effect (γ = 0.416, *p* < 0.001) and indirect effect (γ = 0.034, *p* < 0.05) on QoL. **Conclusions:** These results emphasize the necessity of developing programs aimed at increasing the QoL and mental health of police officers by exploiting these relational dynamics. In addition, the fact that self-esteem played a mediating role in the quality of life among each variable suggests that it is necessary to develop and apply programs to improve self-esteem.

## 1. Introduction

In recent years, Korean citizens have experienced an increase in quality of life (QoL) and well-being, alongside a growing demand for safety. This shift has elevated the social role and expectations placed on police officers. Additionally, the rapid changes and advancements of the times have been associated with increased severity of criminal incidents. In 2022, the crime rate rose to 1952 incidents per 100,000 people, up from 2021 [1], and the number of sexual assault crimes reached 41,433, which was an increase for the second consecutive year [1]. During their duties, police officers are exposed to various traumatic events, including physical threats and violence; they also routinely confront diverse social and natural disasters, such as mass accidents and natural catastrophes [2].

The unpredictable, around-the-clock work environment that police officers experience not only exposes them to physical threats but also places them in a state of psychological instability, such as anxiety and dissociation, and this exposure increases the likelihood that they develop post-traumatic stress disorder (PTSD) [3]. Particularly, police officers show higher rates of PTSD, alcohol abuse, depression, and suicide than the general population [4], and poor mental health can also adversely affect physical health, including diabetes, obesity, and cardiovascular diseases [5]. Thus, the work environment that police officers endure affects not only their physical and mental health but also significantly affects their QoL [6]. In particular, most quality-of-life measurement tools generally categorize and measure physical and mental health into two categories, so physical and mental health are important areas for quality of life [7]. In other words, physical and mental health should be dealt with from a continuous perspective that cannot be separated from quality of life.

Police officers have a legal obligation to respond to work and provide services in a state of tension 24 h a day, and there is a high possibility that their mental health will be harmed due to encountering psychiatric patients in practice or the dangerous environment they experience at work [8]. In particular, when police officers face a crisis, they must solve the problem on their own in an objective and professional manner, and a plan must be prepared to resolve the mental health problems they face through effective communication [9]. In particular, if the mental health of police officers is impaired, it leads not only to physical and mental damage, but also to a decline in professional work performance, and can further cause complex problems beyond personal problems, such as problems with family and drug abuse [9]. Therefore, the mental health of police officers should be examined from a multidimensional perspective as it can have a significant impact on the safety of the nation and its citizens beyond the individual’s problem, and it should be kept in mind that improving the mental health of police officers can ultimately affect the lives of citizens.

The concept of QoL encompasses perceived satisfaction and happiness with one’s current life, which is correlated with psychological well-being; hence, efforts in various dimensions are required to enhance an individual’s QoL [10]. Quality of life is a complex concept that refers to the overall satisfaction of physical, mental, social, and material well-being and is weighted according to the individual’s life values and tendencies [11]. In particular, health is a dimension of quality of life, and health status and clinical symptoms are also recognized as a part of quality of life [12]. Considering that quality of life is subjective satisfaction with life, it must be judged complexly at various levels [13]. This study considered the multidimensional aspects of QoL to identify factors that influence the QoL of police officers by applying the bottom-up spillover theory, which is a component of the spillover theory of happiness [14].

Studies on quality of life are categorized into bottom-up spillover theory, focusing on individual experiences and reactions to external environments, and top-down spillover theory, which highlights inherent individual tendencies [15]. That is, the bottom-up spillover theory states that subjective and cognitive experiences in various areas related to an individual’s life are related to the level of quality of life, while the top-down spillover theory states that the level of quality of life is related to the individual’s characteristics. It is said that the concept of quality of life in bottom-up spillover theory and top-down spillover theory can be determined at a structural level that commonly affects life satisfaction [16].

According to the bottom-up spillover theory [14], the way in which individuals tend to interpret life events and experiences is a consequence of personality traits such as temperament and emotional disposition, and these tendencies affect their assessments of happiness and satisfaction. The top-down spillover theory [14] asserts that objective life conditions serve as primary predictors of overall happiness levels; i.e., individuals are considered to experience happiness as a result of a subjective and cognitive evaluation of emotional characteristics such as satisfaction, pleasure, and joy derived from life events and experiences.

Considering the bottom-up spillover theory, we selected self-esteem as a variable to represent an individual’s personality traits. Self-esteem may encompass an individual’s valuation of their worth, beliefs, and evaluations of their actions [17]. In particular, the bottom-up spillover theory [18] states that in order to satisfy the quality of life, satisfaction with ‘self’ in the life domain must be high. In this study, based on the bottom-up spillover theory, Hypothesis 1 established that self-esteem would have a direct effect on quality of life. Previous studies [19] have demonstrated an association between high self-esteem and high QoL; this observation indicates that self-esteem is a critical variable that affects the QoL of police officers. Research [20] has shown that self-esteem has a positive relationship with sub-domains of psychological well-being, such as autonomy, environmental control, and personal growth. In other words, self-esteem is the foundation of mental health in terms of quality of life and the basis for maintaining quality of life. Another study [21] noted that a positive self-concept, such as self-esteem, is an important factor affecting mental health and psychological well-being. Police officers are exposed to higher stress and physical threats than other occupational groups and are an occupational group that requires not only professional responsibility for maintaining public order and safety but also high self-esteem due to public power [22]. Therefore, self-esteem can be considered a very important variable in the quality of life of police officers in terms of their individual mental health and occupational characteristics more than any other psychological variable.

In the top-down spillover theory, the subjective evaluation of an individual’s emotional characteristics is achieved by quantifying satisfaction; in this study, this condition was operationalized as leisure satisfaction and job satisfaction. From the top-down spillover theory perspective [8], it is said that an individual’s activity and surrounding external environment affect the quality of life. In this study, based on the top-down spillover theory, Hypothesis 2 established that leisure satisfaction has a direct effect on quality of life, and Hypothesis 3 was that job satisfaction had a direct effect on quality of life. In particular, from the perspective of top-down spillover theory, job satisfaction was said to have a positive effect on an individual’s overall life satisfaction, and various activity experiences such as leisure satisfaction were said to be a factor in understanding quality of life [23].

Leisure satisfaction is a positive emotion gained from participating in leisure activities. It focuses on an individual’s positive emotions and states that it is important to utilize leisure effectively in order to be more productive and healthy physically and mentally [24]. Job satisfaction is the satisfaction or dissatisfaction felt with one’s job, and the emotional aspect is emphasized; moreover, it is said to be closely related to the physical and mental well-being of employees [25].

It has been indicated that increased leisure satisfaction positively affects human QoL, including socialization and personal relaxation [26]. The factors that determine QoL depend on how much an individual enjoys their current life [27]. For police officers, if leisure satisfaction is adequately met in daily life, it is considered a significant variable that can enhance QoL, beyond physical and mental health. Job satisfaction is a crucial indicator of a worker’s physical and mental health, and represents the fulfillment of their values, and of their job expectations [28]. High job satisfaction correlates with long tenure, low absenteeism, and increased efforts to increase job performance, and is closely linked to an improved QoL [29].

Therefore, this study focuses on understanding and improving the QoL of Korean police officers who work in a tense and unpredictable environment at various crime scenes.

### Purpose of the Study

The purpose of this study is to investigate the mediating effect of self-esteem on the relationships among job satisfaction, leisure satisfaction, and QoL among Korean police officers. The specific objectives are as follows: (1) Determine the levels of job satisfaction, leisure satisfaction, self-esteem, and QoL among the participants. (2) Assess differences in job satisfaction, leisure satisfaction, self-esteem, and QoL based on the general characteristics of the participants. (3) Examine the correlations among job satisfaction, leisure satisfaction, self-esteem, and QoL among the participants. (4) Identify factors affecting the subject’s quality of life. (5) Identify direct, indirect, and total effects in the path model.

## 2. Materials and Methods

### 2.1. Research Design

This study is a descriptive correlational research investigation that uses mediation analysis to explore the relationships among leisure satisfaction, job satisfaction, QoL, and self-esteem in Korean police officers.

### 2.2. Study Subjects

The subjects of this study were randomly sampled from police officers working in police stations, districts, and outposts within three local police agencies in K Metropolitan City and J Province, Korea. The specific criteria for selecting participants were as follows: (1) They had served as officers in the police department for more than one year. (2) They voluntarily agreed to participate in the study after receiving an explanation about it. Possible subjects were excluded if they were unable to read or communicate in Korean.

To ensure sufficient power for the study, the sample size was calculated using the G*Power 3.1 program for multiple regression, with a significance level (α) of 0.05, power (1 − β) of 0.95, effect size of 0.10, and 12 independent variables, determining a required sample size of 270. Accounting for potential non-returns and disqualifications due to non-responsiveness, 340 questionnaires were distributed. The researchers collected all 340 copies that were distributed. After excluding 10 participants who responded inappropriately to key measures, data from a total of 330 participants were included in the final analysis.

### 2.3. Research Tools

#### 2.3.1. Job Satisfaction

This study used the Korea Minnesota Satisfaction Questionnaire (K-MSQ), which was adapted [30] from the Minnesota Satisfaction Questionnaire (MSQ) [31]. This adaptation has been shown to be both reliable and valid. The K-MSQ consists of 20 items that are designed to measure job satisfaction across three dimensions, which include extrinsic, intrinsic, and general satisfaction. These dimensions are further categorized into three distinct subscales, and each item on the scale is rated on a 5-point Likert scale, where a score of 1 indicates the lowest satisfaction and a score of 5 indicates the highest satisfaction. The overall reliability of this scale, as evidenced by a Cronbach’s alpha of 0.947, was confirmed to be strong in this study.

#### 2.3.2. Leisure Satisfaction

The leisure satisfaction scale (LSS) [32], which has been shown to be reliable and valid [33], was used. The scale includes four main scales: The leisure satisfaction scale includes 24 items that assess six aspects of satisfaction, including psychological, educational, social, relaxation, physical, and aesthetic. Each of these aspects is thoroughly evaluated using a systematic 5-point Likert scale approach, where higher scores signify increased satisfaction. The reliability of this scale in this study was also robust, reflected by a Cronbach’s alpha of 0.970.

#### 2.3.3. Self-Esteem

The self-esteem scale [34,35] was used. This scale is comprised of 10 items, split evenly between five positive and five negative statements, and uses a 5-point Likert scale ranging from 1 (not at all) to 5 (very much so). Both item types are scored similarly, but negative items are reverse-scored during statistical analysis. High scores indicate high self-esteem. In this study, the Cronbach’s α for the scale was 0.780.

#### 2.3.4. Quality of Life

The Korean version of the WHOQOL-BREF [36,37,38] was used. This QoL scale includes 24 items distributed across four domains: eight items assess physical health, four-measure psychological health, six evaluate social relationships, and six appraise environmental factors. The items are rated on a 5-point Likert scale from 1 (not at all) to 5 (very much so). A high score on this scale indicates a higher QoL. In this study, the Cronbach’s α for the scale was 0.930.

### 2.4. Data Collection

This study was conducted with police officers working in three police stations in K Metropolitan City and J Province from 1 to 20 August 2024. The researcher visited the police headquarters, where the purpose of the study was explained to the subjects at each station. Anonymity and confidentiality were assured, and written consent for participation was obtained. Participants were informed that the survey results would be quantified and processed, then used solely for the purposes of this research. They were also assured that their anonymity would be maintained and that they could withdraw from the survey at any time without any negative consequences. The questionnaires were collected immediately after completion; filling them out took approximately 15–20 min. As a token of appreciation, participants were offered a gift.

### 2.5. Data Analysis

Data collected were analyzed using SPSS for Windows, Version 23.0. Descriptive statistics, including frequencies, percentages, means, and standard deviations, were calculated to describe the general characteristics of the participants. Differences in leisure satisfaction, job satisfaction, self-esteem, and QoL were examined using independent *t*-tests and one-way ANOVA, with Scheffé’s test used for post hoc comparisons. Correlations among the main variables were assessed using Pearson’s correlation coefficient r^2^.

Mediation effects were analyzed using PROCESS Macro (Model 6). The significance and pathway differences of the indirect effects were verified using bootstrapping methods to calculate 95% confidence intervals.

## 3. Results

### 3.1. Differences in Job Satisfaction, Leisure Satisfaction, Self-Esteem, and QoL by General Characteristics

Job satisfaction, leisure satisfaction, self-esteem, and QoL were not significantly affected by age, education, or religious affiliation. Marital status had statistically significant effects on both leisure satisfaction and QoL. Years of service significantly affected all measured domains: job satisfaction, leisure satisfaction, self-esteem, and QoL. Job rank significantly affected job satisfaction and QoL. Subjective health significantly affected job satisfaction, leisure satisfaction, self-esteem, and QoL. Economic status was only significantly associated with QoL. Salary satisfaction showed statistically affected job satisfaction and QoL (Table 1).

### 3.2. Correlations Among Study Variables

In this study, QoL was significantly positively correlated with job satisfaction (r = 0.681, *p* < 0.001), leisure satisfaction r = 0.621, *p* < 0.001), and self-esteem (r = 0.443, *p* < 0.001). Self-esteem was also significantly positively correlated with job satisfaction (r = 0.394, *p* < 0.001) and leisure satisfaction (r = 0.364, *p* < 0.001). Leisure satisfaction was significantly positively correlated with job satisfaction (r = 0.470, *p* < 0.001) (Table 2).

### 3.3. Factors Affecting Quality of Life

To investigate the factors influencing the QoL among the participants, nine demographic variables along with the independent variables of self-esteem, leisure satisfaction, and job satisfaction were included in the analysis. Influential factors on QoL were identified as: age > 50 years (β = 0.139, *p* = 0.011), college education level (β = 0.048, *p* = 0.014), medium subjective health status (β = 0.044, *p* = 0.002), job satisfaction (β = 0.039, *p* < 0.001), leisure satisfaction (β = 0.031, *p* < 0.001), and self-esteem (β = 0.035, *p* = 0.015). Together, these variables accounted for 63.4% of the variance in QoL. To assess the correlations among these indices, a multicollinearity analysis was conducted. Multiple regression was used to identify factors influencing the subject‘s QoL.

In this study, the Tolerance Limit ranged from 0.127 to 0.865 and the VIF values were between 1.157 and 7.886; multicollinearity is typically indicated by a Tolerance Limit < 0.1 and a Variance Inflation Factor (VIF) > 10; therefore, these results indicate that multicollinearity did not occur. The Durbin–Watson statistic was 2.113, which confirmed the independence of residuals (Table 3).

### 3.4. Direct Effect, Indirect Effect, and Total Effect in Path Model

Among the factors that affect self-esteem, leisure satisfaction showed statistically significant direct and total effects (γ = 0.199, *p* < 0.001), and job satisfaction also showed statistically significant direct and total effects (γ = 0.293, *p* < 0.001).

**Figure 1:**
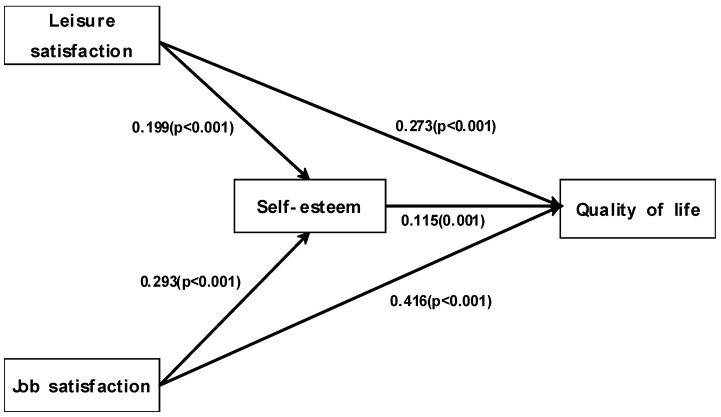
Mediating effects of self-esteem in the relationship between leisure satisfaction, job satisfaction, and quality of life.

Among factors that affect QoL, self-esteem had statistically significant direct and total effects (γ = 0.115, *p* = 0.001). Leisure satisfaction also had significant effects on QoL, both directly (γ = 0.273, *p* < 0.001) and indirectly (γ = 0.023, *p* < 0.05); therefore, its total effect was γ = 0.296 (*p* < 0.001). Job satisfaction had statistically significant direct effects on QoL (γ = 0.416, *p* < 0.001) and indirect effects (γ = 0.034, *p* < 0.05), with a combined total effect of γ = 0.450 (*p* < 0.001) (Table 4). The relationship between leisure satisfaction, job satisfaction, self-esteem, and quality of life is shown in the following figure. Leisure satisfaction, job satisfaction, and self-esteem were shown to have a direct effect on quality of life. Additionally, leisure satisfaction and job satisfaction were found to have a direct effect on self-esteem. Lastly, leisure satisfaction and job satisfaction were found to affect quality of life through the mediating effect of self-esteem (Figure 1).

## 4. Discussion

The purpose of this study was to provide foundational data to support the development of programs designed to enhance the QoL for Korean police officers. It achieved this goal by examining the extent of their life quality and the significance of both direct and indirect effects of factors such as leisure satisfaction, job satisfaction, and self-esteem. The results revealed that both leisure satisfaction and job satisfaction have significant direct and total effects on self-esteem. This finding aligns with research conducted among Portuguese adults [39], which demonstrated that job satisfaction positively influences self-esteem. Similarly, another study [40] found that satisfaction with leisure experiences reduces aggression and has a positive effect on self-esteem. These findings suggest that leisure satisfaction and job satisfaction can improve internal traits and values such as self-esteem, extending beyond the intrinsic behavioral motivation driven by personal interests and satisfaction. This result emphasizes the potential of these factors to activate deep aspects of an individual’s psyche. Future research should analyze satisfaction factors that could increase and promote self-esteem among police officers. Such studies are essential to develop targeted interventions that effectively boost their morale and job performance. Self-esteem has a close impact on satisfaction, and people with high self-esteem are said to have significantly higher satisfaction than people with low self-esteem in terms of personal life satisfaction, satisfaction with interpersonal relationships, physical health, and psychological satisfaction [41]. Previous research [42] on teachers’ self-esteem has shown that teachers with high self-esteem are happier, produce more effective work in the classroom, and judge students more accurately than teachers with low self-esteem. In terms of leisure satisfaction and self-esteem, the satisfaction gained from leisure activities is closely related to an individual’s positive attitude toward leisure, and among various psychological factors, it is closely related to increased self-esteem [43]. Another study [44] also found that self-esteem has a significant effect on satisfaction such as life satisfaction in addition to leisure satisfaction. Since self-esteem is closely related to satisfaction, there is a need to verify satisfaction from various aspects in the future.

The results indicated that self-esteem had both significant direct and total effects on QoL. Research on the effect of self-esteem on QoL has predominantly been conducted among patient populations. For instance, a study involving ostomized patients [45] found self-esteem to be a determinant affecting all facets of their QoL. Similarly, findings from research on healthcare professionals working in high-stress and hazardous environments, analogous to those faced by police officers, show that self-esteem and QoL exert complementary positive influences [46]. Self-esteem is notably effective in influencing professional development [47], and low self-esteem may diminish confidence in high-pressure crime scenes, thereby reducing crisis and problem-solving abilities, and subsequently impeding dedication to professional roles. Moreover, maintaining satisfactory self-esteem can significantly affect personal QoL, and may be a crucial factor that influences overall well-being [48]. The findings suggest that increased self-esteem significantly benefits the QoL among police officers. Future research should focus on developing and applying counseling interventions and self-esteem enhancement programs. In particular, it is necessary to develop a systematic plan to simultaneously improve self-esteem and quality of life through a customized approach that can enhance psychological resilience and self-efficacy.

Leisure satisfaction had significant direct, indirect, and total effects on QoL. This result indicates that while leisure satisfaction directly influences the QoL of police officers, it also indirectly affects it by mediating through self-esteem. Previous research [49] has shown that QoL is increased by satisfaction gained from leisure activities, particularly when psychological satisfaction from leisure is fulfilled. Therefore, although the direct impacts of leisure satisfaction on QoL are significant, fulfilling deeper personal satisfactions, such as self-esteem, also has a strong effect on the improvement of QoL. This observation suggests a need to identify and develop factors that improve the QoL among police officers and to help improve coping mechanisms.

Finally, job satisfaction significantly influenced QoL through direct, indirect, and total effects. Job satisfaction among police officers not only directly affects their QoL but also mediates its effect through self-esteem. Consistent with previous findings [13], job satisfaction is considered a key determinant of QoL. High job satisfaction can reduce the intention to leave the job, increase satisfaction with current life circumstances, reduce workplace stress, and increase QoL. When internal factors like high self-esteem, which can enhance job satisfaction, are present, they strengthen internal motivation. This, in turn, enhances a positive outlook on life and broadens insights into life, thereby positively affecting QoL [39]. Improvements in working conditions and institutional frameworks to boost job satisfaction are crucial; however, low self-esteem not only degrades job satisfaction but also directly reduces QoL. Particularly for police officers working in hazardous and 24-h unpredictable environments, self-esteem is crucial for their professional mission and significantly impacts their QoL. Therefore, ongoing education aimed at maintaining, enhancing, and developing self-esteem is essential.

Although this study did not verify the relationship between job satisfaction and leisure satisfaction, previous studies confirmed that job satisfaction and leisure satisfaction have a positive and significant relationship in psychological and social aspects [24]. Since psychological satisfaction such as quality of life and well-being have a significant impact on human life, future research should continue to seek ways for police officers to maintain a psychosocially stable and healthy life by clearly identifying the relationship between job satisfaction and leisure satisfaction. Meanwhile, a previous study [50] has utilized factors that can be widely used in male-centered daily life, such as alcohol, cigarettes, and physical activity, as factors that affect the quality of life of police officers. In future research, it is believed that setting up more variables that are closely related to real life will be helpful in understanding the impact on the quality of life of police officers in a more practical way. To this end, there is a need to develop a systematic education plan that includes self-esteem improvement workshops, stress management training, and positive psychology-based coaching programs. In addition, it would be effective to introduce case-based problem-solving training and a program to strengthen the support system among colleagues so that it can be used in the actual working environment of police officers.

The limitations of this study are as follows.

First, the research was conducted only among police officers who work at three police stations in K Metropolitan City and J Province, Korea. Therefore, the findings may not be generalizable to all regions of Korea. Moreover, QoL can vary according to circumstances in specific regions and workplaces. Future research should allocate workplaces according to population sizes in different regions to conduct more comprehensive and multidimensional studies.

Secondly, this research was a quantitative study aimed at verifying the mediating effects on the QoL among Korean police officers. Due to its quantitative nature, the study may have limitations in deeply exploring the nuanced aspects of police officers’ QoL. Future research should incorporate qualitative methods to more thoroughly validate the QoL experiences as perceived directly by police officers. Finally, to guide the search for ways to foster both positive physical and mental growth of police officers, the various factors that influence the QoL of Korean police officers must be identified and examined.

Lastly, selection bias is important for errors that occur in selecting or participating in research participants [51]. In other words, the people included in the study and the people appropriate for the study may be different. Although this study involved only those who voluntarily participated in the study, some of them may be forced to do it because others are doing it. This study is a survey study, so the risk of selection bias is less than that of experimental studies. However, future studies should continue to seek ways to minimize selection bias by considering various exogenous factors (working hours, weather, etc.).

Nevertheless, this study is significant because it applied mediation analysis to explore the relationships among leisure satisfaction, job satisfaction, QoL, and self-esteem among a sample of Korean police officers. It successfully identified self-esteem as both a positive internal factor that affects QoL, and as an indirect factor that can increase QoL by mediating various forms of satisfaction. Recently, Korean police officers have faced social disasters, notably the COVID-19 pandemic; therefore, the profession is continually exposed to potential new infections and complex, dangerous situations. Despite the urgent need for research focused on enhancing their physical and mental health within such unpredictable environments, substantial empirical studies remain scarce. This study is significant because it provides foundational data that could facilitate the development of programs and counseling services aimed at improving the QoL for Korean police officers.

## 5. Conclusions

Police officers, as frontline workers in hazardous situations, disproportionately experience post-traumatic stress disorder, physical burnout, and psychological exhaustion compared to other professions. The demanding nature of their roles both diminishes their QoL and heightens their vulnerability to various physical and psychological ailments; the presence of these effects emphasizes the need for continuous institutional reform at the national level.

This study investigated factors that affect the QoL among police officers and analyzed the mediating effects of these factors. The results of this study showed that Leisure Satisfaction had a direct effect on self-esteem and QoL. Job satisfaction had a direct effect on self-esteem and QoL. Self-esteem had a direct effect on QoL. Finally, leisure satisfaction and job satisfaction were found to affect QoL through mediating self-esteem. Factors affecting the quality of life of police officers were identified as age > 50 years, college education level, medium subjective health status, job satisfaction, leisure satisfaction, and self-esteem.

The findings of this study indicate that internal psychological factors like self-esteem have a direct impact on QoL and additionally serve as contributors to personal satisfaction. Consequently, methods to increase self-esteem must be identified. Moreover, because job satisfaction and leisure satisfaction can affect immediate emotions and moods, future research should also consider examining negative internal psychological factors, such as depression and anxiety, as potential mediators of QoL.

In terms of theoretical implications, the quality of life of police officials and the relationship between each variable were confirmed based on bottom-up spillover theory and top-down spillover theory, and directions for promoting the quality of life of police officials were presented. Additionally, in terms of practical implications, it will be necessary to find ways to continuously practice self-esteem in real life through the operation of periodic self-esteem improvement programs and the development of self-esteem tools that reflect the professional characteristics of police officers.

## Figures and Tables

**Table 1:** Distribution by general characteristics of subjects. (N = 330).

Variable	Category	n (%)	Job Satisfaction		Leisure Satisfaction		Self-Esteem		Quality of Life	
			M ± SD, t or F (*p*)		M ± SD, t or F (*p*)		M ± SD, t or F (*p*)		M ± SD, t or F (*p*)	
Age	20s	30 (9.1)	3.29 ± 0.67	2.158 (0.093)	3.64 ± 0.77	0.438 (0.726)	3.60 ± 0.54	0.894 (0.445)	3.43 ± 0.56	1.148 (0.330)
	30s	67 (2.3)	3.22 ± 0.61		3.56 ± 0.69		3.65 ± 0.62		3.33 ± 0.40	
	40s	111 (33.6)	3.26 ± 0.47		3.66 ± 0.57		3.57 ± 0.53		3.35 ± 0.47	
	Over 50s	122 (37)	3.40 ± 0.51		3.67 ± 0.62		3.52 ± 0.53		3.44 ± 0.52	
Marriage	Unmarried a	65 (19.7)	3.33 ± 0.61	0.856 (0.426)	3.68 ± 0.68	5.254 (0.006) c < ba	3.63 ± 0.61	1.718 (0.181)	3.43 ± 0.49	3.605 (0.028) c < ba
	Married b	257 (77.9)	3.31 ± 0.52		3.65 ± 0.62		3.57 ± 0.54		3.39 ± 0.49	
	Etc. c	8 (2.4)	3.07 ± 0.23		2.94 ± 0.37		3.25 ± 0.19		2.95 ± 0.17	
Educational background	High school	59 (17.9)	3.31 ± 0.49	0.461 (0.710)	3.71 ± 0.72	1.038 (0.376)	3.54 ± 0.62	0.104 (0.958)	3.33 ± 0.47	0.545 (0.652)
	College	47 (14.2)	3.27 ± 0.47		3.50 ± 0.61		3.60 ± 0.54		3.34 ± 0.47	
	University	205 (62.1)	3.30 ± 0.54		3.65 ± 0.61		3.57 ± 0.53		3.41 ± 0.48	
	Graduate school or higher	19 (5.8)	3.44 ± 0.79		3.69 ± 0.68		3.54 ± 0.59		3.40 ± 0.68	
Religious affiliation	Yes	116 (35.2)	3.29 ± 0.50	−0.434 (0.665)	3.66 ± 0.59	0.425 (0.661)	3.58 ± 0.52	0.205 (0.838)	3.36 ± 0.44	−0.674 (0.501)
	No	214 (64.8)	3.32 ± 0.56		3.63 ± 0.66		3.57 ± 0.57		3.40 ± 0.51	
Working Period	5 years or less a	42 (12.7)	3.41 ± 0.66	3.242 (0.013) c < ea	3.77 ± 0.74	2.438 (0.047) d < ea	3.80 ± 0.58	3.946 (0.004) c < a	3.51 ± 0.52	5.685 (*p* < 0.001) cd < a
	6–10 years b	60 (18.2)	3.25 ± 0.55		3.52 ± 0.71		3.67 ± 0.50		3.32 ± 0.38	
	11–15 years c	57 (17.3)	3.13 ± 0.46		3.66 ± 0.53		3.42 ± 0.52		3.18 ± 0.39	
	16–20 years d	40 (12.1)	3.25 ± 0.46		3.44 ± 0.48		3.46 ± 0.55		3.29 ± 0.39	
	Over 21 years e	131 (39.7)	3.40 ± 0.52		3.71 ± 0.63		3.55 ± 0.55		3.49 ± 0.55	
Position	Constable a	24 (7.3)	3.43 ± 0.68	6.554 (*p* < 0.001) dcba < e	3.74 ± 0.76	1.717 (0.146)	3.75 ± 0.55	1.06 (0.376)	3.49 ± 0.58	3.534 (0.008) c < ae
	Senior Patrol Officer b	32 (9.7)	3.24 ± 0.57		3.62 ± 0.77		3.61 ± 0.69		3.37 ± 0.44	
	Assistant Inspector c	79 (23.9)	3.21 ± 0.48		3.62 ± 0.61		3.58 ± 0.53		3.27 ± 0.36	
	Inspector d	121 (36.7)	3.21 ± 0.50		3.56 ± 0.62		3.51 ± 0.53		3.35 ± 0.48	
	Senior Inspector or higher e	74 (22.4)	3.56 ± 0.52		3.79 ± 0.57		3.58 ± 0.55		3.54 ± 0.56	
Subjective health status	Upper a	79 (23.9)	3.51 ± 0.65	8.575 (*p* < 0.001) cb < a	3.82 ± 0.66	4.414 (0.013) c < a	3.75 ± 0.65	5.793 (0.003) b < a	3.62 ± 0.52	13.523 (*p* < 0.001) bc < a
	Middle b	227 (68.8)	3.26 ± 0.47		3.60 ± 0.57		3.51 ± 0.50		3.31 ± 0.41	
	Lower c	24 (7.3)	3.13 ± 0.61		3.49 ± 0.94		3.55 ± 0.55		3.36 ± 0.77	
Economic status	Upper a	20 (6.1)	3.50 ± 0.54	2.909 (0.056)	3.76 ± 0.48	0.610 (0.544)	3.42 ± 0.51	1.520 (0.220)	3.53 ± 0.54	3.682 (0.026) c < a
	Middle b	268 (81.2)	3.32 ± 0.53		3.65 ± 0.65		3.59 ± 0.56		3.40 ± 0.49	
	Lower c	42 (12.7)	3.16 ± 0.55		3.57 ± 0.60		3.49 ± 0.51		3.22 ± 0.42	
Salary satisfaction	Upper a	16 (4.8)	3.74 ± 0.72	15.215 (*p* < 0.001) c < b < a	3.85 ± 0.66	2.376 (0.095)	3.50 ± 0.68	0.616 (0.541)	3.64 ± 0.65	1.216 (*p* < 0.001) c < ba
	Middle b	222 (67.3)	3.37 ± 0.50		3.67 ± 0.61		3.59 ± 0.52		3.44 ± 0.50	
	Lower c	92 (27.9)	3.09 ± 0.52		3.54 ± 0.68		3.53 ± 0.60		3.21 ± 0.37	

**Table 2:** Correlation between study variables (n = 330).

	Job Satisfaction	Leisure Satisfaction	Self-Esteem	Quality of Life
	r (*p*)	r (*p*)	r (*p*)	r (*p*)
Job satisfaction	1			
Leisure satisfaction	0.470 (*p* < 0.001)	1		
Self-esteem	0.394 (*p* < 0.001)	0.364 (*p* < 0.001)	1	
Quality of life	0.681 (*p* < 0.001)	0.621 (*p* < 0.001)	0.443 (*p* < 0.001)	1

**Table 3:** Factors affecting quality of life.

Factors	B	β	SE	t	*p*
(Constant)	0.752	0.174		4.324	*p* < 0.001
Age (ref: 20 s)					
30 s	−0.117	0.106	−0.097	−1.102	0.271
40 s	−0.200	0.126	−0.195	−1.587	0.114
Over 50 s	−0.355	0.139	−0.353	−2.545	0.011
Marriage (ref: Unmarried)					
Married	−0.008	0.065	−0.007	−0.126	0.900
Etc	−0.112	0.129	−0.036	−0.869	0.386
Educational background (ref: High school)					
College	0.071	0.061	0.051	1.166	0.244
University	0.118	0.048	0.118	2.473	0.014
Graduate school or higher	−0.019	0.083	−0.009	−0.235	0.815
Religious affiliation (ref: Yes)					
No	0.048	0.036	0.048	1.325	0.186
Working Period (ref: 5 years or less)					
6–10 years	0.004	0.097	0.003	0.039	0.969
11–15 years	−0.082	0.120	−0.064	−0.684	0.495
16–20 years	0.066	0.121	0.045	0.546	0.586
Over 21 years	0.250	0.128	0.253	1.949	0.052
Position (ref: Constable)					
Senior Patrol Officer	0.059	0.108	0.036	0.546	0.585
Assistant Inspector	0.047	0.128	0.042	0.368	0.713
Inspector	0.102	0.132	0.101	0.769	0.442
Senior Inspector or higher	0.050	0.138	0.043	0.361	0.718
Subjective health status (ref: Upper)					
Middle	−0.138	0.044	−0.132	−3.155	0.002
Lower	0.056	0.076	0.030	0.735	0.463
Economic status (ref: Upper)					
Middle	0.013	0.085	0.010	0.152	0.880
Lower	−0.076	0.101	−0.052	−0.749	0.455
Salary satisfaction (ref: Upper)					
Middle	0.095	0.095	0.092	1.005	0.316
Lower	−0.002	0.100	−0.002	−0.019	0.985
Self-esteem	0.086	0.035	0.098	2.445	0.015
Leisure satisfaction	0.277	0.031	0.362	8.975	*p* < 0.001
Job satisfaction	0.387	0.039	0.429	9.987	*p* < 0.001

Durbin–Watson = 2.113, Tolerance Limit = 0.127~0.865, VIF = 1.157~7.886, R^2^ = 0.663, Adjusted R^2^ = 0.634, F = 22.876, *p* < 0.001.

**Table 4:** Direct Effect, Indirect Effect, and Total Effect in path model.

Variables	Categories	Direct Effect (*p*)	Indirect Effect (*p*)	Total Effect (*p*)
Self-esteem	Leisure satisfaction	0.199 (*p* < 0.001)		0.199 (*p* < 0.001)
	Job satisfaction	0.293 (*p* < 0.001)		0.293 (*p* < 0.001)
Quality of life	Self-esteem	0.115 (0.001)		0.115 (0.001)
	Leisure satisfaction	0.273 (*p* < 0.001)	0.023 (*p* < 0.05)	0.296 (*p* < 0.001)
	Job satisfaction	0.416 (*p* < 0.001)	0.034 (*p* < 0.05)	0.450 (*p* < 0.001)

## Data Availability

The original contributions presented in the study are included in the article; further inquiries can be directed to the corresponding author.

## References

[1] Statistics Korea (2024). Korea Safety Report 2023. www.kostat.go.kr.

[2] Violanti J.M., Charles L.E., McCanlies E., Hartley T.A., Baughman P., Andrew M.E., Fekedulegn D., Ma C.C., Mnatsakanova A., Burchfiel C.M. (2017). Police stressors and health: A state-of-the-art review. Policing.

[3] Wagner S.L., White N., Fyfe T., Matthews L.R., Randall C., Regehr C., White M., Alden L.E., Buys N., Carey M.G. (2020). Systematic review of posttraumatic stress disorder in police officers following routine work-related critical incident exposure. Am. J. Ind. Med..

[4] Trombka M., Demarzo M., Campos D., Antonio S.B., Cicuto K., Walcher A.L., Garcia-Campayo J., Schuman-Olivier Z., Rocha N.S. (2021). Mindfulness training improves quality of life and reduces depression and anxiety symptoms among police officers: Results From the POLICE study—A multicenter randomized controlled trial. Front. Psychiatry.

[5] Heyman M., Dill J., Douglas R. (2018). The Ruderman White Paper on Mental Health and Suicide of First Responders.

[6] Wu X., Liu Q., Li Q., Tian Z., Tan H. (2019). Health related quality of life and its determinants among criminal police officers. Int. J. Environ. Res. Public Health.

[7] Ginieri-Coccossis M., Triantafillou E., Tomaras V., A Liappas I., Christodoulou G.N., Papadimitriou G.N. (2009). Quality of life in menta lly ill, physically ill and healthy individuals: The validation of the Greek version of the World Health Organization Quality of Life (WHOQOL-100) questionnaire. Ann. Gen. Psychiatry.

[8] Lamb H.R., Weinberger L.E., DeCuir W.J. (2002). The police and mental health. Psychiatr. Serv..

[9] Kelley T.M. (2005). Mental health and prospective police professionals. Polic. Int. J..

[10] Hajiran H. (2006). Toward a Quality of life Theory: Net Domestic Product of Happiness. Soc. Indic. Res..

[11] Felce D., Perry J. (1995). Quality of life: Its definition and measurement. Res. Dev. Disabil..

[12] Ferrans C.E. (1990). Quality of Life: Conceptual Issues. Semin. Oncol. Nurs..

[13] Andresen I.H., Hansen T., Grov E.K. (2017). Norwegian nurses’ quality of life, job satisfaction, as well as intention to change jobs. Nord. J. Nurs. Res..

[14] Diener E. (1984). Subjective well-being. Psychol. Bull..

[15] Sirgy M.J. (2001). Handbook of Quality-of-Life Research: An Ethical Marketing Perspective.

[16] Sirgy M.J., Efraty D., Siegel P., Lee D.J. (2001). A new measure of quality of work life (QWL) based on need satisfaction and spillover theories. Soc. Indic. Res..

[17] Hunt O.T., Johnston C.D., Hepper P.G., Burden D.J. (2001). The psychosocial impact of orthognathic surgery: A systematic review. Am. J. Orthod. Dentofacial. Orthop..

[18] Ghyas Q.M., Kondo F. (2015). The Contribution of Mobile Information Services to Improve the Quality of Young User’s Lives Based on Bottom-Up Spillover Theory: A Case Study on Japan.

[19] Johal A., Alyaqoobi I., Patel R., Cox S. (2015). The impact of orthodontic treatment on quality of life and self-esteem in adult patients. Eur. J. Orthod..

[20] Paradise A.W., Kernis M.H. (2002). Self-esteem and psychological well-being: Implications of fragile self-esteem. J. Soc. Clin. Psychol..

[21] Langeveld N.E., Grootenhuis M.A., Voute P.A., De Haan R.J., Van Den Bos C. (2004). Quality of life, self-esteem and worries in young a dult survivors of childhood cancer. Psycho-Oncol. J. Psychol. Soc. Behav. Dimens. Cancer.

[22] Lipp M.E.N. (2009). Stress and quality of life of senior Brazilian police officers. Span. J. Psychol..

[23] Massam B.H. (2002). Quality of life: Public planning and private living. Prog. Plann..

[24] Koç M.C., Er Y. (2020). Leisure Satisfaction and Job Satisfaction: A Research on Academics. Afr. Educ. Res. J..

[25] Kurt N., Demirbolat A.O. (2019). Investigation of the relationship between psychological capital perception, psychological well-being and job satisfaction of teachers. J. Educ. Learn..

[26] Argan M., Argan M.T., Dursun M.T. (2018). Examining relationships among well-being, leisure satisfaction, life satisfaction, and happiness. Int. J. Med. Res. Health Sci..

[27] Rajani N.B., Skianis V., Filippidis F.T. (2019). Association of environmental and sociodemographic factors with life satisfaction in 27 European countries. BMC Public Health.

[28] AL-Dossary R., Vail J., Macfarlane F. (2012). Job satisfaction of nurses in a Saudi Arabian university teaching hospital: A cross-sectional study. Int. Nurs. Rev..

[29] Atefi N., Abdullah K.L., Wong L.P. (2016). Job satisfaction of Malaysian registered nurses: A qualitative study. Nurs. Crit. Care.

[30] Park S.Y., Kim J.H. (2019). Campus life adaptation scale for nursing undergraduates: Development and psychometric evaluation. Nurs. Educ. Today.

[31] Dawis R.V., Lofquist L.H. (1984). A Psychological Theory of Work Adjustment.

[32] Beard J.G., Ragheb M.G. (1980). Measuring leisure satisfaction. J. Leis. Res..

[33] Kim M.L., Lee Y.J., Hwang S.H. (2010). Cross-cultural validation test and application of LSS-short form. J. Korea Contents Assoc..

[34] Rosenberg M. (1965). Society and Adolescent Selfimage.

[35] Jeon B.J. (1974). Self-esteem: A test of its measurability. Yonsei Nonchong.

[36] World Health Organization (1996). WHOQOL-BREF: Introduction, Administration, Scoring and Generic Version of the Assessment—Field Trial Version.

[37] Min S.K., Lee C.I., Kim K.I., Suh S.Y., Kim D.K. (2000). Development of Korean Version of WHO Quality of Life Scale Abbreviated Version (WHOQOL-BREF). Psychiatry Investig..

[38] Kang S.T. (2012). The Relationship Between the Restriction on Leisure, Lifestyle and the Quality of Life on a Police and a Fire-Fighting Officer Who Is Participated in Leisure Activity. Ph.D. Thesis.

[39] Satuf C., Monteiro S., Pereira H., Esgalhado G., Marina Afonso R., Loureiro M. (2018). The protective effect of job satisfaction in health, happiness, well-being and self-esteem. Int. J. Occup. Saf. Ergon..

[40] Xia X., Wang X., Yu H. (2022). Mediating effect of self-esteem on the relationship between leisure experience and aggression. Sci. Rep..

[41] Brown J.D. (1998). The Self.

[42] Reilly E., Dhingra K., Boduszek D. (2014). Teachers’ self-efficacy beliefs, self-esteem amd job stress as determinants of job satisfaction. Int. J. Educ. Manag..

[43] Kim S., Sung J., Park J., Dittmore S.W. (2015). The relationship among leisure attitude, satisfaction, and psychological wellbeing for college students. J. Phys. Educ. Sport..

[44] Choi S., Yoo Y. (2017). Leisure attitude and satisfaction with leisure and life: Proposing leisure prioritization and justification. World Leis. J..

[45] Ferreira E.D.C., Barbosa M.H., Sonobe H.M., Barichello E. (2017). Self-esteem and health-related quality of life in ostomized patients. Rev. Bras. Enferm..

[46] Franjić D., Franjić I., Babić D., Grgić S., Spahalić M. (2021). Association of resilience with quality of life and selfesteem in healthcare workers of covid-19 hospital. Zdravstveni Glasnik..

[47] Sodeify R., Moghaddam T.F. (2020). Nursing students’ perceptions of effective factors on mental health: A qualitative content analysis. Int. J. Community Based Nurs. Midwifery.

[48] Park S.A., Jung S.H., Park H.S. (2019). The Effects of Stress, Selfesteem, and Resilience on Nursing Student’s Quality of Life. J. Humanit. Soc. Sci..

[49] Zhou B., Zhang Y., Dong E., Ryan C., Li P. (2021). Leisure satisfaction and quality of life of residents in Ningbo, China. J. Leis. Res..

[50] Costa F.G.D., Vieira L.S., Cócaro M.G., Azzolin K.D.O., Dal Pai D., Tavares J.P. (2020). Quality of life, health conditions and life style of civil police officers. Rev. Gaúcha Enferm.

[51] Tripepi G., Jager K.J., Dekker F.W., Zoccali C. (2010). Selection bias and information bias in clinical research. Nephron. Clin. Pract..

